# Towards Easy-to-Use Bacteria Sensing: Modeling and Simulation of a New Environmental Impedimetric Biosensor in Fluids

**DOI:** 10.3390/s21041487

**Published:** 2021-02-21

**Authors:** Christian Pfeffer, Yue Liang, Helmut Grothe, Bernhard Wolf, Ralf Brederlow

**Affiliations:** 1 Department of Electrical and Computer Engineering, Technical University of Munich, 80333 Munich, Germany; ge29qeq@mytum.de (Y.L.); grothe@tum.de (H.G.); r.brederlow@tum.de (R.B.); 2 Steinbeistransferzentrum für Medizinische Elektronik und Lab-on-Chip Systeme, 80802 Munich, Germany; wolf@tum.de

**Keywords:** biosensor, FEM simulation, sensor design, electrochemical sensor, bacteria detection, bacteria sensor, pathogens, impedance spectroscopy

## Abstract

Conventional pathogenic bacteria-detection methods are lab-bound, time-consuming and need trained personnel. Microelectrodes can be used to recognize harmful microorganisms by dielectric impedance spectroscopy. However, crucial for this spectroscopy method are the spatial dimensions and layout of the electrodes, as the corresponding distribution of the electric field defines the sensor system parameters such as sensitivity, SNR, and dynamic range. Therefore, a variety of sensor models are created and evaluated. FEM simulations in 2D and 3D are conducted for this impedimetric sensor. The authors tested differently shaped structures, verified the linear influence of the excitation amplitude and developed a mathematical concept for a quality factor that practically allows us to distinguish arbitrary sensor designs and layouts. The effect of guard electrodes blocking outer influences on the electric field are investigated, and essential configurations are explored. The results lead to optimized electronic sensors in terms of geometrical dimensions. Possible material choices for real sensors as well as design and layout recommendations are presented.

## 1. Introduction and State of the Art

Pathogenic bacteria represent a prime threat to medical facilities because of their fast growth and harmful effects on human health [1]. Conventional detection methods require time-consuming processes including sensing of characteristic metabolites or cellular reproductive cycles. For decades, researchers have been looking for fast and accurate detection and identification systems for bacterial strains [2]. Recent advances in micro- and nano-fabrication technologies have led to significant improvement of detection limits, and reduced the time required for sample preparation [3].

Impedance Spectroscopy (IS), or Electrochemical Impedance Spectroscopy (EIS), can be used to detect bacteria in liquids [4,5,6]. Specifically, it is of interest to attract bacteria to the surface of an electrode via an applied field, and its attractive force to a polarizable cell. However, crucial for impedance spectroscopy method and for effective attraction are the spatial dimensions and layout of the electrodes, as the corresponding distribution of the electric field highly defines the sensor system behavior. Even though many experimental approaches have been taken [7,8,9,10,11], the precise underlying concepts to EIS bacterial detection have not yet been investigated thoroughly by numerical approaches.

Here, a systematic study on bacterial, electrochemical impedimetric sensors is presented. After briefly illuminating other methods for biofilm or cellular lifeform detection, the work focuses on design aspects that are essential for the further development of biosensors of this kind. In general, the investigation comprises two steps. First, the attraction of bacteria to the sensor surface by DC potentials is explored. Second, classical impedance spectroscopy for detection is the method of choice for read-out. The goals are to illuminate and verify the physical concepts behind the sensor, including an optimized attraction method for cells in fluids, as well as finding the best geometry. The role of electrode periodicity is addressed, and a new mathematical tool for evaluation of arbitrary, complex sensor models is presented. Finally, novel layout and design recommendations for future biosensors are given.

The state of the art divides into the two phases of operation before reviewing the landscape of bacterial sensors. First, the attraction of bacteria and their adhesion on the sensor surface is considered. Applying DC potentials on electrodes to move and attract microorganisms in solution has been scientifically proven [7,8,9,10].

Next, bacterial detection via EIS (using AC potentials) is presented in a series of projects. A recent review describes the literature around pathogen detection very well [11]. In general, the use of microfabrication of bacterial sensors has made it possible to integrate multiple processes in sequence for one-step sensing or in parallel for high-throughput screening [3,12].

Lab-on-chip provides a convenient, cost-effective approach that is compatible with microfluidics. In addition, electrical methods do not require a labeling step for sensing target bacteria. Boehm et al. constructed a microfluidic bacteria sensor based on measuring the impedance in a fixed-volume chamber containing cells [13]. Platinum microelectrodes are used to discriminate two bacterial strains *E. coli* and M.catarrhalis in a few minutes using a change in impedance. By contrast, Carbonaro et al. have developed an on-chip artificial pore that could be used to detect bacterial pathogens [14,15].

Cheng et al. presented a microfluidic system out of two parallel glass slides and a thin PDMS gasket [16]. After cells were adhered to the glass surface and modified with proteins specific to the target cells, they were lysed to monitor a change in conductivity that correlates with the number of cells. A lower detection limit was presented as 20 cell/uL [3]. Electrochemical impedance spectroscopy is a label-less sensing technique. Liao et al. developed a microfluidic electrochemical sensor array for detection of uropathogens in human clinical fluid [17]. In line with the development of impedance-based sensors, the electrical impedance output can be further amplified by a parallel set of electrode configurations such as interdigitated array microelectrodes (IDA). Many parallel electrodes and a large active surface area improve the detection limit and response time [3,18]. Laczka et al. report a bacterial cell detection limit of 1500 cells/mL [18]. Lei et al. took a capacitive approach [3,19]. Yang et al. applied IDA microelectrodes for the detection of variable Salmonella Typhimurium in milk. The microelectrodes, consisting of indium-tin-oxide (ITO), measure an impedance change during bacterial cell growth [20]. A bacterial suspension with the initial concentration of $10^{5}$ CFU/mL could be detected in 2.2 h. In contrast to the previous approaches Lu et al. have detected bacterial cells in an insulating environment using IDA gold electrodes. The inter-electrode spacing was further reduced to 2 μm in this device, enabling an attachment of single bacteria across two adjacent electrodes. The current was closely associated with the number of bacterial cells that formed a conducting bridge between adjacent electrodes. Although the sensor is extremely sensitive at detecting a single bacterium, it requires a clear understanding of the conducting mechanism of the bacterial cells on the electrode surface [3].

Other essential approaches in pathogen detection are nanomaterials, such as nanoparticles, nanotubes, nanowires and nanomechanical switches. Current nano-fabrication technology can make the size of a sensing probe comparable to those of bacteria or other target pathogens, improving the sensitivity and detection limit of a sensor [21,22]. Elkin et al. coated the carbon nanotube surfaces with bovine serum albumin (BSA) to improve the solubility of the nanotubes in aqueous solution, and further constructed nanotube-protein conjugates with pathogen-specific antibodies to detect *E. coli* O157:H7. Direct measurement of conductance between two electrodes with a nano-sized gap can also be a highly sensitive technique for biosensing purpose. The applications have been extended to detect a pathogen cell bigger than small biomolecules, such as virus or antigens [3,11].

Several groups have demonstrated a highly sensitive immunosensor based on a field effect transistor (FET) constructed with nanotubes [22,23]. Optical approaches involving surface plasmons can also be found [24].

With the maturity of the current nano-fabrication technology, the fabrication and assembly of the new sensors has become a straightforward process. However, reliability, repeatability, durability, and ease of operation are important issues for lab-on-a-chip pathogen sensors. In addition, the integration of a complex process within a single chip still faces challenges [3]. As geometrical parameters highly influence the sensor performance [18], FEM simulations started to foster the understanding of this kind of biosensor [25].

## 2. Materials and Methods

The AC DC Module of COMSOL Multiphysics© v5.5 is used for FEM Simulations [26]. To evaluate arbitrary structures, a quality factor is defined. The quality factor is based on the electric field norm of the gradient. The quality factor needs precise volumes of interest, specified in Section 4.5 for further reading.

An optical Zeiss Metallux microscope is used to obtain photographs of fabricated structures.

## 3. Theory

COMSOL© provides a numerical solution solving the underlying Maxwell equations for the desired model. First, the scalar field of the electric potential is calculated. The second derivative of the potential gives the electric field gradient $V_{ij}$, see Equation (1).

(1) $$V_{ij}=\frac{\partial^{2}\;V}{\partial x_{i}\partial x_{j}}$$

The length of a gradient is defined via the norm in Equation (2). In the case of a polarizable bacterium in an electric field, the gradient corresponds to a dielectric mechanical force acting on it.

(2) $$\left|\right|V_{ij}\left|\right|=\sqrt{\Delta E_{x}\cdot \Delta E_{x}+\Delta E_{y}\cdot \Delta E_{y}+\Delta E_{z}\cdot \Delta E_{z}}$$

Directed towards to electrodes that span the electric field, these forces shall be maximized in the later application by creating fields as inhomogeneous as possible.

**Figure 1:**
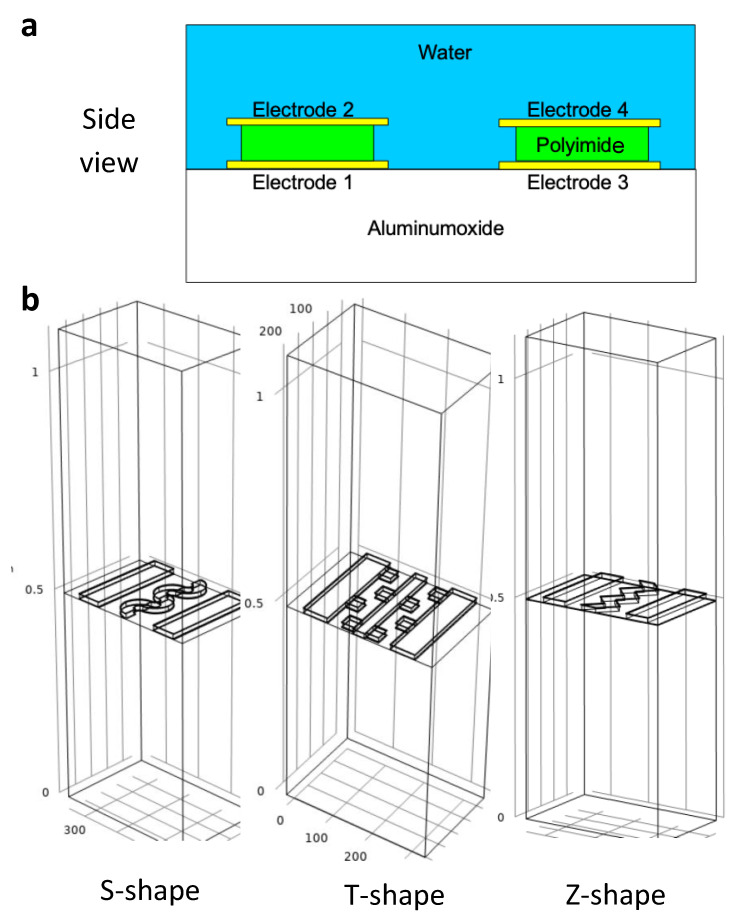
(**a**) Electrode configuration and used nomenclature in this work. Polyimide acts as an insulator between the electrodes (**b**) Designed 3D structures for Gathering and Detection of Bacteria

The electrode configurations and their nomenclature can be seen in Figure 1. It also presents the main 3D models that will be investigated in further detail in the further sections.

Electrochemical effects such as the pH effect must be taken into account for real-life applications. This work assumes small applied potentials, low surface area of the electrodes and the low ionic strength that are expected to lead to very low involved currents and by that to very low chemical reactions on the electrodes surface.

## 4. Results

### 4.1. Concept and Verification of Bacteria Detection

Dielectric or Electrochemical Impedance Spectroscopy relies on changes of the dielectric properties of the surrounding materials. The method is used as a read-out. Low concentrations of bacteria are to be expected in most applications. Hence, an attraction phase is also included in the investigations. In this phase, polarizable bacteria can be attracted by high field gradients that directly lead to a dielectric force pushing the cells closer to the sensor’s electrode. Further details are discussed in Section 4.3.

**Figure 2:**
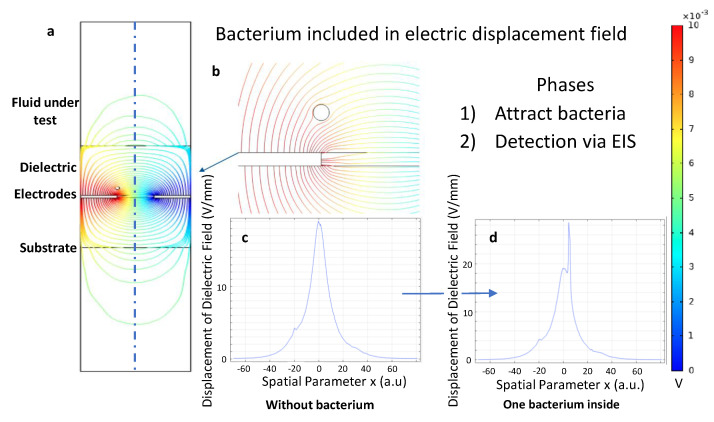
Concept of Bacteria Interfering with the Displacement Field: (**a**) A 2D two-electrode simulation of the displacement field when applying an external potential. (**b**) A circular model of a bacteria interacting with the displacement field between the electrodes. First, bacteria re attracted by applying DC potentials to the electrodes. In a second step, the read-out follows via electrochemical impedance spectroscopy (EIS) using AC potentials. (**c**,**d**) Displacement of the electric field in the center of the model in 1D with and without bacteria. The plot is evaluated along the dashed line in the model, starting from the center as indicated in (**a**). The color scale indicates low potentials (blue) to high potentials (red).

The color scale indicates low (blue) and high (red) potentials in the following plots. In Figure 2, a sensor stack is simulated with Finite-Element Method. Between the electrodes a dielectric displacement field arises when a potential of 10 mV is applied. Going along the dashed line, starting from the center of the model, a total displacement can be calculated. The spatial parameter in Figure 2c,d is based on the dashed line and peaks in the middle of the electrodes in Figure 2a. The total displacement shows significant changes when a circular model of a bacterium exists somewhere in the sensor stack. The parameters of the bacteria model have been chosen equivalently to the properties of *E. coli*.

### 4.2. Material Choice

**Figure 3:**
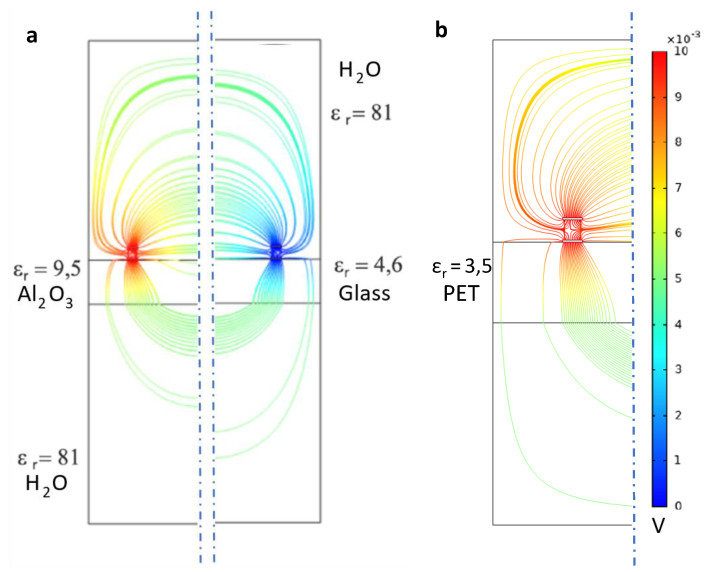
FEM Electric Field Simulations for Substrate Material Selection. (**a**) Solid substrates. The left side indicates aluminum oxide. The right-hand side shows the electrical field for borosilicate glass. (**b**) Flexible Substrate PET (Polyethylene Terephthalate). Corresponding dielectric constants have been added to the desired parts. The color scale indicates low potentials (blue) to high potentials (red).

A major advantage of modern FEM simulations is the ability to change the involved materials, or rather material properties of the objects in the model. Rapid prototyping massively benefits as the materials can be tested in the simulations. A 2D simulation is performed for this purpose and satisfies the required level of complexity. In Figure 3, the two-electrodes model from Figure 2 is used to study possible substrates with different permittivity. In this setup, the electrical field lines are symmetrical. Thus, the plot is split in the middle for two different substrates, aluminum oxide and borosilicate glass. As substrate material Al${}_{2}$O${}_{3}$ and borosilicate glass are used due to their low dielectric dissipation factor and their insolubility in an aqueous environment. Also, a flexible substrate PET (Polyethylene Terephthalate) is presented. As the electrical field lines can change when the substrate is deformed, a rigid substrate is preferred. The electrodes are fabricated out of gold.

Bacteria shall be gathered to the proximity of the sensor’s electrodes. Aluminum oxide as the substrate proves more concentrated field lines which also makes the mechanical force much stronger. From this simulation plot, aluminum oxide should be applied as the material in the sensor structure design.

### 4.3. 3D Electrode Designs

**Figure 4:**
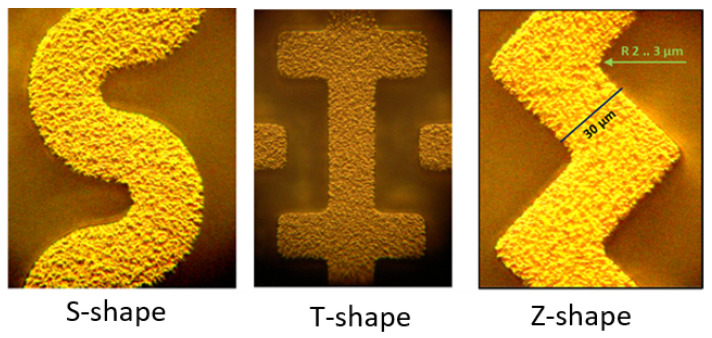
Optical micrographs of fabricated structures based on the models in the theory section and Figure 1.

The goal of this work is to be able to evaluate arbitrary electrode structures for the purpose of detecting bacteria. However, the complexity of the models, especially in 3D, is quite high. As an analytical solution is very challenging, the work tests and evaluates models that can be fabricated as well. In Figure 4 three sensor models of electrodes in 3D are shown in an example of fabrication. Picking the right dimensions depends on available fabrication methods, and hence defines the minimal spatial resolution. The used dimensions can be realized as shown in photographs taken on an optical microscope. Au has been used as an electrode. Lithographic smearing effects have been simulated briefly, but seemed to be neglectable to a reasonable extent.

Experimental proof of fabrication is taken with an optical microscope.

The concentrations of bacteria in fluids are quite low, even if biofilms form. It is worthwhile to note that a detection limit is $10^{4}$ bacteria per ml. [4]. A miniaturized sensor is a more economical approach. The trade-off is a smaller area where the sensor can interact with the bacteria. To overcome this limit, two phases of operation are realized (see also Figure 2).

First, there is the physical attraction or gathering of the bacteria. The authors expect high field gradients that can polarize a bacterium, and therefore, a mechanical force in the direction of the electrodes act on them. The second phase is the detection when a critical concentration of bacteria is in the vicinity of the electrodes. The physical signal of the attendance of the desired bacteria can be realized with Impedance Spectroscopy in the real-world application [4]. It is important to the sensor layer design that it should have enough force to attract the bacteria in the sensor layer. The Z-shape has regular straight edges and right angles which are easily manufactured and produced. This shape could maximize the adsorption of bacteria on the surface of the sensor; even the bacteria at the corner could be affected.

### 4.4. Periodicity of Multiple Electrodes

**Figure 5:**
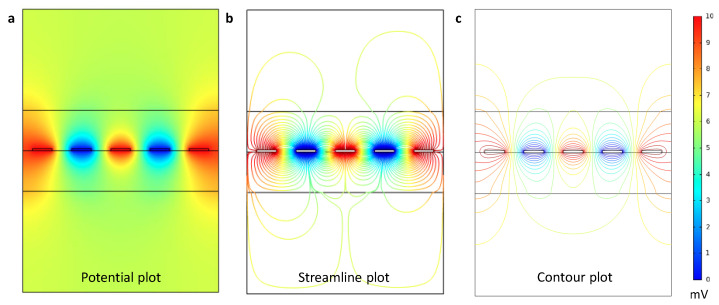
Periodicity of Multiple Electrodes: (**a**) Potential plot of five electrodes with two outer guard electrodes. (**b**) Streamline plot showing electrical field lines and possible asymmetries. (**c**) Contour Plot. The color scale indicates low potentials (blue) to high potentials (red) but is reversible for the purpose on estimating field distributions. The stack follows the same partitioning as in Figure 2.

By working on geometrical parameters, the authors put effort into arranging the field lines that suit the purpose of bacterial attraction and detection. External fields play an important role in their impact on the internal field lines and their shielding, respectively. As the electrical field can be disturbed from outside of the sensor, a possible array of electrodes is investigated in Figure 5. One approach is to increase the number of electrodes to an arbitrary number. The outer electrodes can be used as guard electrodes when putting ground potential on them. Again, the reduction to 2D is sufficient here and simplifies the issue.

For this purpose, external fields are minimized through the guard electrodes and show the extent of the displacement field in the outer versus the inner electrodes. The guard electrodes have an influence on both modes of operation. On the one hand, bacterial attraction benefits from shielding external fields. On the other, impedance spectroscopy relies on optimized internal fields. Therefore, significant sensor performance parameter improvements, such as a better signal-to-noise ratio, can be achieved this way.

### 4.5. Definition of Quality Factor in 3D

**Figure 6:**
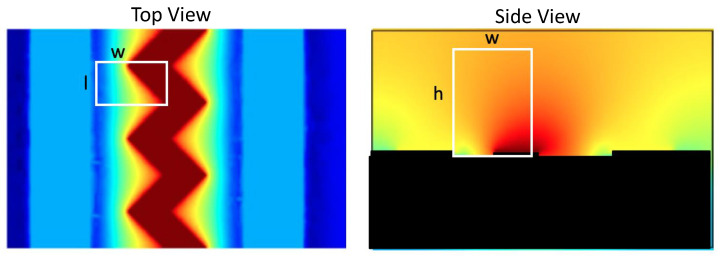
Towards full mathematical evaluation of 3D simulations: Definition of quality factor. COMSOL Multiphysics© gives a numerical solution to the Maxwell equations. The potential in each single point of the 3D model is calculated and can be used to derive electrical gradients in the mesh. W represents the distance from line contact edge to middle of zigzag contact; I is the distance from peak to valley; and h is the height of interest volume.

The geometry of the sensor design defines the ability to gather bacteria. The attraction of bacteria needs optimized geometrical parameters; however, the qualitative evaluation of arbitrary models is challenging as the whole volume above the sensor is important for the purpose. To create a metric to measure the quality of each sensor structure, a quality factor is defined. The goal here is to derive a metric that not only evaluates the fields in a single point or line, but instead over the whole simulated space. Hence, a volume of interest is defined as in Equation (3) and visualized in Figure 6. The spatial parameters used are the length (*l*), width (*w*) and height (*h*).

(3) $$V_{interest}=l\cdot w\cdot h$$

Basically, starting from the potential all gradients are calculated to indicate the corresponding attractive force on polarizable objects in the fluid. A 3D grid with the mesh size of 1 μm is defined as volume of interest. This allows the division of the complete simulation space into smaller pieces. The gradient in each of these boxes are geometrically summed up and divided again by the by the volume of interest.

Higher gradients lead to stronger force while the gradients are distributed over the active sensor area. Therefore, the sum of these gives an indication on the effect. The second step—the detection via EIS—also benefits indirectly from this metric. As more bacteria are in the very proximity of the sensors the available sensor performance increases.

### 4.6. Full Evaluation of 3D Models, the Role of Configurations and Excitation Amplitude

**Figure 7:**
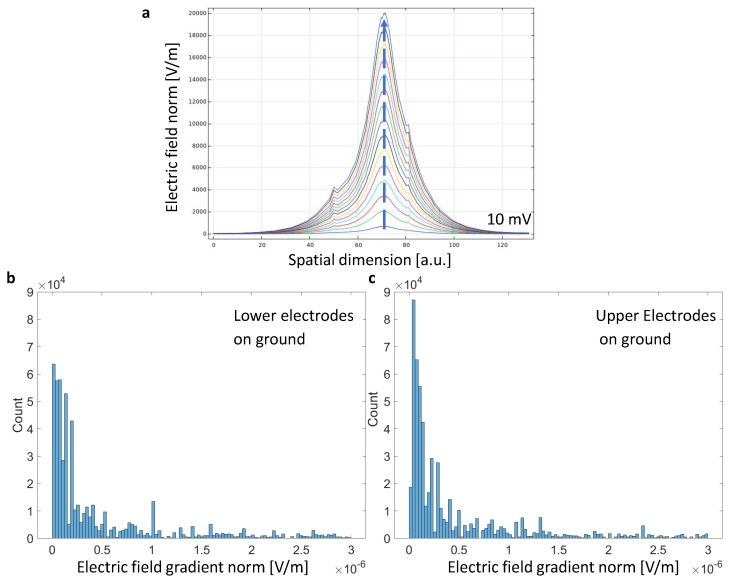
Distributions of Gradient Lengths across Volume of Interest, the Influence of Configuration of the Electrodes and the Role of Excitation Amplitude: (**a**) The role of excitation amplitude for the gradient length ranges from 10 mV (bottom line) to 300 mV (step size 20 mV in a single, center point of the model. (**b**) Using the concept of the quality factor a histogram depicts the full 3D distributions of gradient lengths in the simulated model ZF. The electrodes are configured in way that the bottom ones directly on the substrate are on ground. (**c**) Switching the configuration on the same model exhibits a different configuration.

The excitation signal varies the maximum electric field norm in the very center of the models; see Figure 7a. The spatial dimension refers to the 2D line as in Figure 2. The center peaks plotted go from 10 mV to 300 mV in 20 mV step size appear to go linearly with the electric field norm. The two smaller side peaks are due to material interfaces, e.g., substrate to fluid. It is worth noting that the excitation amplitude has deliberately not exceeded 300 mV. The bare electrodes are partly in contact with the fluid and it is advisable to stay outside the electro-active window.

More importantly, not only 2D but 3D data is gained and evaluated by the concept of the quality factor. Figure 7b,c shows the results on the distribution of gradient lengths in the full model volume. Remarkably, choosing the electrodes to different configurations leads to distinguished outcomes. Quantization errors occur in the numerical approach and challenge the search on an adequate fit function for arbitrary models. However, using the quality factor and then applying the arithmetic mean on the histogram showed promising results. The configuration of setting the lower electrodes to ground was superior to all other configurations, see Table 1. A higher quality factor can be interpreted as larger, average gradients in the overall volume units of the model.

This approach allows for mathematical analysis of the FEM models in addition to a visual evaluation of the structures.

### 4.7. Sensor Layer Geometry Comparison between T-Shape, S-Shape, Z-Shape and ZF-Shape

**Figure 8:**
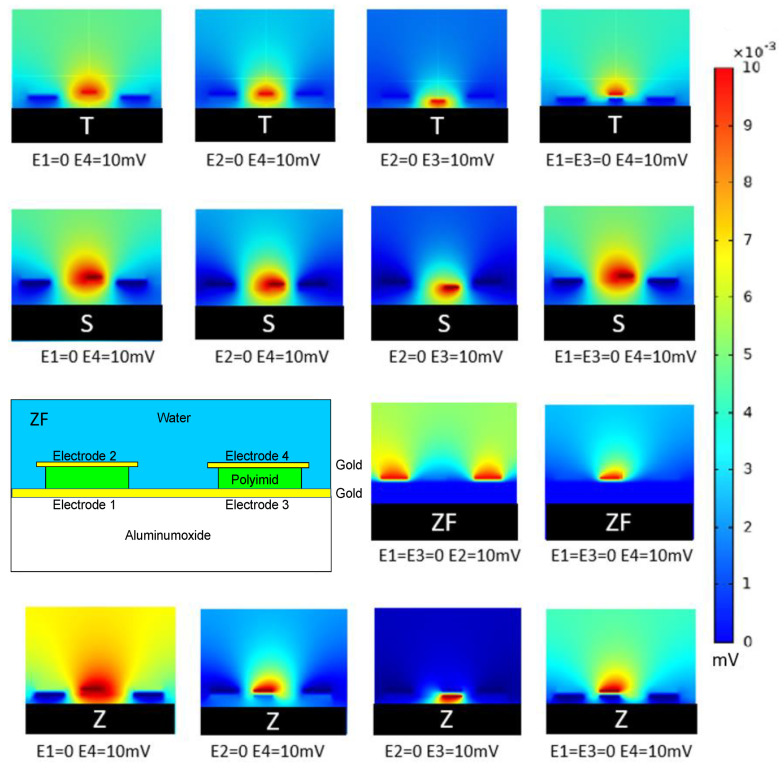
Comparison of Field Distributions on T-,S-, Z and ZF-shaped 3D Structure of the Sensor Layer. A modified version of the Z-model with connected bottom electrodes is included, called ZF-structure. A four-electrode setup is used for shielding purposes. The nomenclature of the electrode configurations is given on the left side and shows the big impact on how the potentials are applied to each electrode. Unmentioned electrodes are kept on floating potential. The color scale indicates low potentials (blue) to high potentials (red).

To find the most efficient structure to attract the microorganisms, four kinds of structures have been designed and simulated to find out their potential distributions. One structure, ZF, is shown in Figure 8, but is exactly like the Z-structure except of one minor difference. The bottom electrodes are connected to each other in the ZF-structure. Side views of the best-performing models can be seen in Figure 8.

Simulated potential plots with different combinations of electrode potentials are compared with a focus on field distributions in the central region. Among these combinations, ‘E4’ is applied with 10 mV, and ‘E2’ or ‘E1 = E3’ regarded as ground; these combinations have much higher gradient distribution visible even in optical inspection.

A compilation of the results when applying the quality factor to the models can be found in Table 1. The combination which shows higher gradient distribution is selected and is chosen to be further developed experimentally. From the many combinations more were tested; however, the ones with at least one bottom electrode (E4) performed best.

In general, the modified version of the presented Z-structure with connected bottom electrodes (ZF) achieves the highest ranking of all in terms of large areas with high inhomogeneities for bacterial attraction (DC potentials).

## 5. Discussion

For the purpose of attracting and detecting microorganisms in solutions, a conceptual study is presented. Starting from the physical background, applied mathematical concepts are used to consider the real-life sensing framework (e.g., electrode configurations). This work aims to broaden the understanding of impedimetric biosensors using numerical simulations.

The change of the displacement field on polarizable models of bacteria is investigated in Figure 2. A simulational proof of concept for movement of bacteria in electrical fields is presented. As the typical particle size of *E. coli* is in the micrometer-range, diffusional forces acting on the bacteria are neglected [27]. The conducted FEM simulations are used to derive the best materials for practical sensors as seen in Figure 4. This work demonstrates the positive role of guard electrodes in Figure 5. An efficient shielding can be realized by adding additional, constantly biased electrodes.

The mathematical construct of quality factor works for arbitrary models. Starting from the numerical solution to the Maxwell equations, this work successfully evaluated S-, T-, and Z-shaped designs. Sharp edged Z and ZF- structures are superior as they provide a higher field gradient distribution to gather microbes close to the electrodes. To compare arbitrarily, the work shows that FEM simulations allow deep insights in the field distributions. Regarding the two phases of bacteria attraction and detection, conclusions on the acting forces can be drawn (see Figure 2) [7]. This work defines a quality factor for bacteria attraction which can simplify the selection of optimal field distributions in Figure 7. These FEM simulations are used to select optimal materials and sensor topologies for our fabricated sensors. Two-electrode sensors are likely to perform worse than three- and four-electrode setups. Regarding how to configure or connect the electrodes for a real sensor, a strong recommendation from the simulation result is to set the substrate electrodes to ground, as shown in Figure 8. A superior sensor performance is expected when the full potential drop lays in the active sensor area. However, this figure is not straightforward to interpret. Hence, the authors developed the quality factor. Table 1 summarizes the results and ranks the tested structures.

Compared to the state of the art, this theoretical study shows that more complex electrode setups can improve both accumulation of bacteria at the surface and therefore also the sensitivity of the sensor. This work contributes to the scientific community by providing a mathematical tool for evaluation and clear recommendations for design and layout of next-generation biosensors.

## 6. Conclusions

An in-depth analysis of 2D and 3D electrode structures and their behavior when performing electrochemical impedance spectroscopy for bacteria attracting and sensing purposes has not been done to the best of the author’s knowledge. Reliability, repeatability, durability, and ease of operation are important issues for lab-on-a-chip pathogen sensors and heavily rely on the sensor layout and design. The integration of a complex process within a single chip still faces challenges [3,22,23]. Towards the realization of a real-world application, electrochemical effects such as the pH effect must be taken into account. This work contributes to overcome these hurdles and sets a theoretical framework for further investigations.

In particular, this study can be used to fabricate optimized electrode structures for bacterial sensing in fluids. Crucial design parameters in Impedance Spectroscopy for bacteria sensors are researched. Specifically, the role of guard electrodes on microelectrodes, the excitation amplitude, and a mathematical tool to evaluate sensors are presented.

In times of a pandemic, and the known lifespan of thin biofilms in dry and liquid state is an interesting parameter to eventually detect pathogens in public spaces. Label-free electrochemical sensing can be an inexpensive possibility for tracking whether cleaning is needed. Another interesting route can be the digestion of pH-insensitive, cost-effective sensors. As the human metabolism is still a great mystery in many aspects, pills using this technology might be able to help identify certain cell cultures in the digestion track. This kind of biosensor represents an alternative route to enzymatic sensor approaches.

## Figures and Tables

**Table 1:** Quality Factors for various sensor designs. Not mentioned electrodes are kept on floating potential.

Type	Electrode Configuration	QF	Ranking
S	E1 = E3 = 0 E4 = 10 mV	1.28014	7
S	E2 = 0 E4 = 10 mV	1.66557	6
T	E1 = E3 = 0 E4 = 10 mV	1.95682	4
T	E2 = 0 E4 = 10 mV	2.35999	3
Z	E1 = E3 = 0 E4 = 10 mV	1.83386	5
Z	E2 = 0 E4 = 10 mV	2.93997	2
ZF	E1 = E3 = 0 E4 = 10 mV	3.23390	1

## Data Availability

The data presented in this study are available on request from the corresponding author.

## Abbreviations

The following abbreviations are used in this manuscript:

DC	Direct Current
AC	Alternating Current
IDA	Interdigitated Electrode Array
EIS	Electrochemical Impedance Spectroscopy
FEM	Finite-Element Method

## References

[1] Jamal M., Ahmad W., Andleeb S., Jalil F., Imran M., Nawaz M.A., Hussain T., Ali M., Rafiq M., Kamil M.A. (2018). Bacterial biofilm and associated infections. J. Chin. Med. Assoc..

[2] Ivnitski D., Abdel-Hamid I., Atanasov P., Wilkins E. (1999). Biosensors for detection of pathogenic bacteria. Biosens. Bioelectron..

[3] Heo J., Hua S.Z. (2009). An overview of recent strategies in pathogen sensing. Sensors.

[4] Bernhard Gleich T.W. (2004). Mikrosensor-System zur Bestimmung des Kontaminationsgrades von Oberflächen. Abschlussbericht Mikrobe FöRderkennzeichen 16SV1117/9.

[5] Schwarz M., Jendrusch M., Constantinou I. (2020). Spatially resolved electrical impedance methods for cell and particle characterization. Electrophoresis.

[6] Weyh T., Wendicke K., Gleich B., Wolf B. (2002). Optimisation of Bacteria Sensitive Sensor. IFMBE Proceedings.

[7] Koyama S., Konishi M.a., Ohta Y., Miwa T., Hatada Y., Toyofuku T., Maruyama T., Nogi Y., Kato C., Tsubouchi T. (2013). Attachment and detachment of living microorganisms using a potential-controlled electrode. Mar. Biotechnol..

[8] Saavedra A., García-Meza J.V., Cortón E., González I. (2020). Attachment of Leptospirillum sp. to chemically modified pyrite surfaces. Fast and simple electrochemical monitoring of bacterial-mineral interactions. Hydrometallurgy.

[9] Koyama S., Nishi S., Tokuda M., Uemura M., Ishikawa Y., Seya T., Chow S., Ise Y., Hatada Y., Fujiwara Y. (2015). Electrical retrieval of living microorganisms from cryopreserved marine sponges using a potential-controlled electrode. Mar. Biotechnol..

[10] Koyama S., Yoshida T. (2016). Electrical Collection of Membrane-intact and Dehydrogenase-positive Symbiotic Bacteria from the Deep-sea Bivalve Calyptogena Okutanii. Electrochemistry.

[11] Cesewski E., Johnson B.N. (2020). Electrochemical biosensors for pathogen detection. Biosens. Bioelectron..

[12] Deisingh A.K., Thompson M. (2002). Detection of infectious and toxigenic bacteria. Analyst.

[13] Boehm D.A., Gottlieb P.A., Hua S.Z. (2007). On-chip microfluidic biosensor for bacterial detection and identification. Sens. Actuators B Chem..

[14] Carbonaro A., Mohanty S.K., Huang H., Godley L.A., Sohn L.L. (2008). Cell characterization using a protein-functionalized pore. Lab Chip.

[15] Raymundo-Pereira P.A., Shimizu F.M., Coelho D., Piazzeta M.H., Gobbi A.L., Machado S.A., Oliveira O.N. (2016). A Nanostructured Bifunctional platform for Sensing of Glucose Biomarker in Artificial Saliva: Synergy in hybrid Pt/Au surfaces. Biosens. Bioelectron..

[16] Cheng X., Liu Y.S., Irimia D., Demirci U., Yang L., Zamir L., Rodriguez W.R., Toner M., Bashir R. (2007). Cell detection and counting through cell lysate impedance spectroscopy in microfluidic devices. Lab Chip.

[17] Liao J.C., Mastali M., Gau V., Suchard M.A., Moller A.K., Bruckner D.A., Babbitt J.T., Li Y., Gornbein J., Landaw E.M. (2006). Use of electrochemical DNA biosensors for rapid molecular identification of uropathogens in clinical urine specimens. J. Clin. Microbiol..

[18] Laczka O., Baldrich E., Munoz F.X., del Campo F.J. (2008). Detection of Escherichia coli and Salmonella typhimurium using interdigitated microelectrode capacitive immunosensors: The importance of transducer geometry. Anal. Chem..

[19] Yao L., Hajj-Hassan M., Ghafar-Zadeh E., Shabani A., Chodavarapu V., Zourob M. CMOS capactive sensor system for bacteria detection using phage organisms. Proceedings of the 2008 Canadian Conference on Electrical and Computer Engineering.

[20] Yang L., Li Y., Griffis C.L., Johnson M.G. (2004). Interdigitated microelectrode (IME) impedance sensor for the detection of viable Salmonella typhimurium. Biosens. Bioelectron..

[21] Elkin T., Jiang X., Taylor S., Lin Y., Gu L., Yang H., Brown J., Collins S., Sun Y.P. (2005). Immuno-carbon nanotubes and recognition of pathogens. ChemBioChem.

[22] Lin Y., Jiang X., Elkin T., Fernando K., Gu L., Taylor S., Yang H., Jones E., Wang W., Sun Y.P. (2006). Carbon nanotubes for immunomagnetic separation of Escherichia coli O157: H7. J. Nanosci. Nanotechnol..

[23] Seo S., Kim H.C., Cheng M., Ruan X., Ruan W. (2006). Microelectrical noise detector for rapid, specific, and sensitive identification of bacteria. J. Vac. Sci. Technol. B Microelectron. Nanometer Struct. Process. Meas. Phenom..

[24] Béland P., Krupin O., Berini P. (2015). Selective detection of bacteria in urine with a long-range surface plasmon waveguide biosensor. Biomed. Opt. Express.

[25] Olmo A., Yúfera A. Computer Simulation of Microelectrode based Bio-Impedance Measurements with COMSOL. Proceedings of the Biodevices.

[26] COMSOL, C.M AC/DC Module User’s Guide COMSOL Multiphysics^®^v. 5.5. COMSOL. COMSOL Multiphysics^®^ v. 5.5. COMSOL AB.

[27] Adamczyk Z. (2013). Diffusion of Particles. Encyclopedia of Colloid and Interface Science.

